# The role of nonhuman primate models in the development of cell-based therapies for Parkinson’s disease

**DOI:** 10.1007/s00702-017-1708-9

**Published:** 2017-03-22

**Authors:** Scott C. Vermilyea, Marina E. Emborg

**Affiliations:** 1 Neuroscience Training Program, University of Wisconsin, Madison, 1220 Capitol Court, Madison, WI 53715 USA; 2 Wisconsin National Primate Research Center, University of Wisconsin, Madison, USA; 3 Department of Medical Physics, University of Wisconsin, Madison, USA

**Keywords:** Parkinson’s disease, Nonhuman primates, Dopamine, Stem cells

## Abstract

Through the course of over three decades, nonhuman primate (NHP) studies on cell-based therapies (CBTs) for Parkinson’s disease (PD) have provided insight into the feasibility, safety and efficacy of the approach, methods of cell collection and preparation, cell viability, as well as potential brain targets. Today, NHP research continues to be a vital source of information for improving cell grafts and analyzing how the host affects graft survival, integration and function. Overall, this article aims to discuss the role that NHP models of PD have played in CBT development and highlights specific issues that need to be considered to maximize the value of NHP studies for the successful clinical translation of CBTs.

## Introduction

Since the early 1980s, scientists have relied on nonhuman primate (NHP) models to assess whether cell-based therapies (CBTs) can be beneficial for Parkinson’s disease (PD). CBT strategies and NHP models of PD emerged into the scientific arena simultaneously. In a way, the availability of the new NHP models fueled CBT progression towards clinical application.

**Fig. 1:**
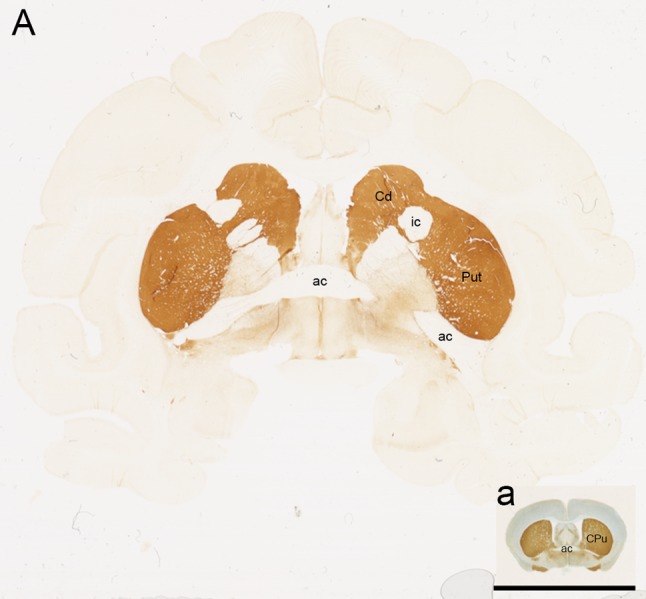
Coronal brain sections of **A** rhesus and *a* mouse brain immunostained against tyrosine hydroxylase (TH) highlighting the comparison of brain size and complexity. *Scale bar* 1 cm. *Cd* caudate, *ic* internal capsule, *Put* putamen, *ac* anterior commissure, *CPu* caudate and putamen

As the main aim of CBTs for PD was and still is the replacement of neurons lost in the disease, PD animal models with neurotoxin-induced neuronal loss became an ideal platform to assess the approach. CBT studies in rodent models provided invaluable information on neuronal survival, migration and integration after grafting (Kim et al. 2013). Clinical translation of CBTs requires progressive evaluation in different species and as a first-in-class and invasive brain therapy, NHP experiments are a logical next step (Capitanio and Emborg 2008). Compared to rodents that are inbred, NHPs are outbred. Behavioral outcome measures such as fine motor skills, which are affected in PD, can be easily tested in NHPs but not in other large species, like pigs. Clinically relevant behavioral outcome measures are critical to determine the efficacy of the strategy, including the selection of intracerebral grafting targets. In that regard, NHPs and humans share a similar organization of the striatum, with the caudate nucleus and the putamen clearly delineated by the white matter tracts of the internal capsule. In rodents, transecting white matter tracts perforate throughout the striata, without presenting a physical barrier for cell distribution (Fig. 1).

In this article, we aim to discuss the role of NHP models of PD in CBT development, keeping in perspective how the field of PD is evolving, analyze ongoing advances in CBTs and the issues that need to be considered to maximize the value of NHP studies for the successful clinical translation of CBTs for PD.

## Parkinson’s disease: then and now

Parkinson’s disease (PD) is the 2nd most common neurodegenerative disorder affecting around 1% of the population over the age of 60 (Driver et al. 2009). When CBTs were first envisioned for actual clinical application, the conceptualization of PD suggested that it was an ideal candidate disease for brain repair. Patients were diagnosed by typical motor symptoms (resting tremor, rigidity, bradykinesia, and postural instability), which were associated with the loss of dopaminergic (DAergic) nigral neurons. As the symptoms responded to oral dopamine (DA) replacement therapy, it was expected that dopamine replacement with a cell source should be an efficient way to securely and locally deliver DA and, basically, cure PD. In addition, the brain was viewed as an immunoprivileged, postmitotic organ.

The understanding of PD and the brain has evolved over time, which affects the application of CBT strategies. Today, PD diagnoses still depend on the presence of typical motor symptoms, and postmortem confirmation of nigral DAergic neuron loss and the presence of intracytoplasmic neuronal inclusions termed Lewy bodies [LBs; (Vermilyea and Emborg 2015)]. Yet, PD is now recognized as a complex neurodegenerative disorder that includes non-motor symptoms (NMS). Depression, anxiety, loss of sense of smell, gastrointestinal dysfunction and cardiac dysautonomia are common PD NMS, which are associated with neurodegeneration in other areas of the central and peripheral nervous system (Chaudhuri and Odin 2010; Chaudhuri et al. 2011). Interestingly, NMS precede the onset of the movement disorder by decades and are now proposed as prodromal signs of the disease (Postuma et al. 2012). Earlier PD diagnoses would increase the chances of success of neuroprotective strategies. In that regard, brain immunoreactivity has been documented (Kordower et al. 1997; Roitberg et al. 2004; Tambur 2004) and neuro-inflammation has been linked to PD neurodegeneration, suggesting that immunomodulation can be neuroprotective (Kannarkat et al. 2013). Neurogenesis has been documented in the adult brain of rodents (Altman and Das 1965; Kaplan and Hinds 1977; Kempermann et al. 1997), NHPs (Gould et al. 1999) and humans (Eriksson et al. 1998), and directed neurogenesis has been discussed for self-brain repair (Rakic 2004).

Another chain of events that led to findings with great implications for PD and CBTs started in 1996, when mutations in the alpha-synuclein (α-syn) gene were found in familial forms of PD (Polymeropoulos et al. 1997). Subsequent studies identified α-syn as the main component of LBs (Spillantini et al. 1997, 1998). Then, in 2008 LBs were reported in dopaminergic fetal grafts of PD patients that were transplanted a decade earlier, suggesting that the grafts “caught PD” from the host (Li et al. 2008; Kordower et al. 2008). Since then, α-syn research has taken a center stage in PD research (Bendor et al. 2013; Vermilyea and Emborg 2015). Investigations on whether α-syn has prion-like activity revitalized the Braak and Braak hypothesis that PD may start in the brainstem and propagate through the neural axis (Braak et al. 2004; Chu and Kordower 2015; Hilker et al. 2011). Studies on protein aggregation followed (Luk et al. 2009), as well as the search for neuroprotective approaches aiming to prevent aggregation (Kalia et al. 2015). It should be noted that the cause of PD is still unclear and that the question of whether the early peripheral symptoms reflect where PD starts or less neuroplasticity is being debated (Engelender and Isacson 2016).

## NHP models of PD used for CBT evaluation

Common marmoset, vervet and macaque monkeys are the most used NHP species for CBT studies. To the best of our knowledge, only neurotoxin-induced NHP models of PD have been used as testing platforms for CBTs, mainly by the administration of 6-hydroxydopamine (6-OHDA) or 1-methyl-4-phenyl-1,2,3,6-tetrahydropyridine (MPTP), although new models are emerging (Table 1).

**Table 1:** Comparison between NHP models of PD highlighting key features for evaluation of CBTs for DA cell replacement

NHP PD model	PD motor symptoms	Nigrostriatal DA depletion	Typical LIDs	Synucleinopathy	Comments
6-OHDA	Yes	Yes	No	Not reported	Unilateral model. Requires multiple intracerebral stereotaxic injections to induce a stable lesion
MPTP systemic	Yes	Yes	Yes	Upregulation of a-syn	Bilateral model. Needs to be individually titrated, and depending on dosing paradigm may require from one week to over a year to induce syndrome. PD symptoms may spontaneously recover
MPTP systemic+aging	Yes	Yes	Yes	Upregulation of α-syn, possible aggregates	Same as MPTP systemic plus animals may require more intensive care post intoxication
MPTP ICA	Yes	Yes	No	Not reported	Unilateral model. Requires surgical set up; Induces a stable and reproducible lesion
MPTP ICA+aging	Yes	Yes	No	Not reported	Same as MPTP ICA except MPTP dose needs to be 2/3 of younger animals. Monkeys may require more intensive care post intoxication
Aged NHPs	Yes	Yes	No	Translocation of α-syn	Not enough dopamine deficit to be responsive to l-DOPA treatment
Viral vector delivery of α-syn	Yes (Common marmosets)	Yes	Unknown	Overexpression of α-syn, aggregates	Unilateral model. Requires intracerebral stereotaxic injections
Lewy body extracts	No	Yes	Unknown	Overexpression of α-syn, aggregates	Unilateral model. Requires intracerebral stereotaxic injections
α-syn Transgenic	Mild (Cynomolgus 1.5–2 years old)	Unknown	Unknown	Unknown	Bilateral model

*6-OHDA* 6 hydroxydopamine, *α-syn* alpha-synuclein, *MPTP* 1-methyl-4-phenyl-1,2,3,6-tetrahydropyridine, *ICA* intracarotid artery, *l* *-DOPA* L-3,4-dihydroxyphenylalanine, *LIDs* l-DOPA induced dyskinesias

6-OHDA (Senoh and Witkop 1959) is preferentially used in common marmoset monkeys (Ungerstedt 1968; Blandini et al. 2008; Eslamboli et al. 2005). As 6-OHDA does not cross the blood–brain barrier (BBB), it is stereotaxically injected directly into the right or left striatum, medial forebrain bundle or substantia nigra, inducing unilateral motor impairments. The neurotoxin is selectively taken up by catecholaminergic neurons through monoamine transporters, and induces sympathetic neuronal loss by increasing the production of reactive oxygen species (ROS), and disrupting energy metabolism and neuronal activity (Blum et al. 2001).

MPTP (Davis et al. 1979; Langston et al. 1983) is most commonly administered to macaque monkeys, although it is also used in other old and new world NHP species (Fox and Brotchie 2010; Emborg 2007). Unlike 6-OHDA, MPTP crosses the BBB and is administered to NHPs via s.c., i.m., i.v. or intracarotid artery (ICA) injection. Severity of PD symptoms depends on route, dose and frequency of administration; dosing regimen varies between species (Emborg 2007). In the brain, MPTP is transformed into its toxic metabolite MPP^+^ by monoamine oxidase-B (MAO-B). MPP^+^ is then selectively taken up by the DA transporter into dopaminergic neurons where it disrupts normal mitochondrial respiration by acting as a mitochondrial complex I inhibitor, leading to oxidative stress and apoptosis (Przedborski and Vila 2001).

Although it can be argued that neurotoxic models are missing critical components of PD, such as “true” LB formation (Dauer and Przedborski 2003), several reasons justify their use to test CBTs as DA replacement/network restoration strategies: (1) neurotoxin-induced models present PD-like motor symptoms and dopaminergic nigrostriatal loss, (2) they are well characterized, and (3) they can be induced in a protracted period of time, which facilitates their use as testing platforms.

We agree with the concept that an ideal NHP model of PD should mimic the disease by replicating its etiology, which should induce the pathological mechanisms that give rise to the typical symptoms. Yet, although great progress has been made towards understanding the complexity of PD and possible pathways of neurodegeneration, its cause is still unknown. Neurotoxin-based models attempt to capitalize on the knowledge that exposure to environmental toxins is a PD risk factor. In that regard, both neurotoxins trigger mechanisms associated with neurodegeneration in PD. In addition to disrupting energy metabolism and increasing oxidative stress, dosing with 6-OHDA and MPTP induces inflammatory responses (Rodriguez-Pallares et al. 2007; Joglar et al. 2009; McGeer et al. 2003). MPTP dosing has also been shown to trigger an increase in α-syn expression and, in some cases, its accumulation (Halliday et al. 2009; Kowall et al. 2000; McCormack et al. 2008).

Other identified PD risk factors include aging and genetic mutations which are exploited for modeling purposes (Emborg 2007). Studies in aged NHPs with or without MPTP have been reported. Intact aged animals present subtle PD-like symptoms (e.g.: slowness and overall decreased amount of movement) and nigrostriatal dopaminergic loss, with individual variations. As the symptoms in aged monkeys do not respond to DA replacement therapies, the animals are not useful models to assess cell replacement strategies. In contrast, aged animals intoxicated with MPTP present the typical motor and pathological syndrome observed after neurotoxin, plus the background aged condition associated with PD, and could be useful testing platforms, yet CBT studies in models combining aging and neurotoxins have not been reported.

Genetic-like NHP PD models have been induced by intracerebral injection of viral vectors encoding for mutated α-syn or administration of LB extracts [see review (Vermilyea and Emborg 2015)]. Adeno-associated viral (AAV) vector-induced nigral overexpression of human α-syn wild type and A53T has been shown to induce PD-like motor symptoms, significant nigral dopaminergic cell loss, and α-syn aggregates in common marmoset monkeys (Eslamboli et al. 2007; Kirik et al. 2003). AAV and lentiviral vectors encoding for A53T α-syn have also been used in cynomolgus (Koprich et al. 2016) and rhesus (Yang et al. 2015) monkeys. In both studies, A53T α-syn induced nigral cell loss and α-syn accumulation and aggregation; behavioral changes were not reported. A combination of AAV-induced overexpression of Parkin and A53T α-syn was reported in cynomolgus; although the animals had decreased striatal dopaminergic markers and α-syn accumulation and phosphorylation, no motor symptoms were observed. It should be noted that in vervet monkeys nigral injection of AAV expressing a short hairpin RNA (shRNA) to knock down α-syn induced a region-specific decrease in TH-positive nigral cell number and striatal innervation compared to animals that received scrambled shRNA; no behavioral changes were reported (Collier et al. 2016). Intracerebral inoculation of α-syn fibrils has been extensively used in rodents, but not yet in monkeys (e.g.: (Luk et al. 2012; Paumier et al. 2015)). Instead cadaveric LB extracts have been injected into the striatum or nigra of cynomolgus monkeys with or without previous MPTP, (Recasens et al. 2014). The extracts induced some decreases in striatal and nigral dopaminergic markers and increases in α-syn expression, yet PD motor symptoms were not detected. It should be noted that with exception of the AAV α-syn studies in marmosets, all the other reports in genetic-like models were performed in a few subjects; further characterization and validation of the models are needed before they are used as testing platforms for CBTs. Transgenic NHP models induced by oocyte injection of lentiviral vectors encoding for mutations of interest are emerging; transgenic rhesus monkeys overexpressing mutant A53T α-syn have been reported (Niu et al. 2015). The authors also reported some behavioral deficits after 1.5–2.5 years of age. New technologies such as CRISPR/Cas9 genomic editing present an opportunity to create NHP models with PD associated mutations expressed at physiological levels that may help clarify the disease onset process, including motor and non-motor symptoms (Gaj et al. 2016). Timely evaluation of CBTs in these novel NHP models may provide clues to understand α-syn-related problems during clinical translation and define the role of CBTs in global therapies.

## CBTs for DA cell replacement: from rodents and monkeys to PD patients

Fetal mesencephalon and autologous adrenal medullary tissues were the first sources used to demonstrate the feasibility for DA cell replacement. In 1979, Bjorklund and Stenevi (Bjorklund and Stenevi 1979) reported positive effects of fetal grafts in circling behavior of 6-OHDA-treated rats, and in 1981, Freed et al. (Freed et al. 1981) showed adrenal graft survival in a similar model. In 1984, Morihisa and colleagues (Morihisa et al. 1984) transplanted adrenal medullary tissue and fetal mesencephalic cells into MPTP-treated parkinsonian rhesus monkeys and showed some survival of adrenal, but not fetal, cells. In follow-up experiments, poor survival of transplanted adrenal tissue was reported (Morihisa et al. 1987) yet as the PD signs ameliorated, researchers hypothesized that the intense host-derived dopaminergic sprouting in the transplanted area was responsible for the behavioral improvements. Improved graft survival was observed when methods were applied to minimize the endothelial components (Schueler et al. 1993). With regard to fetal mesencephalic grafts, between 1986 and 1994, 15 NHP reports were published (Bakay et al. 1987; Collier et al. 1987; Redmond et al. 1986, 1988; Sladek et al. 1986; Annett et al. 1990, 1994; Bankiewicz et al. 1990; Collier et al. 1994; Dubach et al. 1988; Elsworth et al. 1994; Fine et al. 1988; Sladek et al. 1993; Taylor et al. 1990, 1991) demonstrating the feasibility of the approach, as well as different degrees of antiparkinsonian effects. It should be noted that these studies were performed in a limited number of NHPs per treatment group, in most cases the area of the brain targeted was the caudate nucleus, the animals did not receive immunosuppression and the effect of antiparkinsonian medication was not evaluated (Fitzpatrick et al. 2009).

Both CBT approaches were rapidly translated to humans. In 1985, Backlund et al. reported two cases of patients receiving adrenal medulla grafts resulting in mild effects, and in 1987, Madrazo and colleagues showed the first dramatic improvement of PD symptoms in two patients (Madrazo et al. 1987). Following these promising results, several similar case studies were undertaken, with different, less favorable outcomes [see review: (Redmond 2002)]. Goetz et al. reported the results of a multi-center study of 19 patients in 1989 (Goetz et al. 1989). The grafted patients showed minimal temporary antiparkinsonian effects, described as decreased mean severity of “off” time (time when the positive effects of pharmacological treatment wear off) as assessed by both the Activities of Daily Living subscale of the Unified Parkinson’s Disease Rating Scale (UPDRS) and the Schwab and England scale. Yet, the patients’ antiparkinsonian medications could not be decreased and postoperative morbidity was considerable, due to the double surgery (abdominal and brain) required to harvest the adrenal medulla and then transplant the cells. Postmortem results revealed poor cell survival and localized host regional neuronal sprouting, similar to the results in the NHP experiments.

With regard to fetal mesencephalon grafting, after multiple case reports in PD patients [see (Freed et al. 1992; Redmond et al. 1993)] the National Institute of Health (NIH) funded two prospective, double blind, randomized control trials aiming to assess the efficacy and safety of transplanting fetal mesencephalic tissue to treat PD. The Freed et al. (Freed et al. 2001) trial consisted of 40 PD patients: 20 received bilateral post-commissural putaminal grafts of ventral mesencephalic fetal neurons, and 20 had a burr hole drilled into their skull as a sham procedure. Immunosuppression was not administered. The fetal cells were cultured for 1 week prior to transplantation. The primary endpoint was patient self-reports on activities of daily living (recorded at home for 12 months). The Olanow et al. trial (2003) consisted of 34 PD patients: 11 received bilateral post-commissural putaminal grafts of ventral mesencephalic fetal neurons from one donor fetus, 12 from four donor fetuses, and 11 received bilateral sham procedures. Oral cyclosporine (CsA) immunosuppression was administered to all patients starting 2 weeks prior to surgery and continued for 6 months after grafting. The primary endpoint was the UPDRS motor subscore. Both trials did not show significant differences between treatment groups, although further analysis revealed that patients younger than 60 years old or with less severe PD at baseline had improvements in their parkinsonian signs. Positron Emission Tomography (PET) imaging demonstrated increased [^18^F]fluorodopa uptake in the grafted areas suggesting graft survival that was later confirmed by postmortem examinations.

An unexpected outcome for both trials was the occurrence of what was then named “runaway” dyskinesias, also known as graft-induced dyskinesias (GIDs). Unlike typical PD dyskinesias that are induced by chronic long-term l-DOPA administration [see (Bezard et al. 2001)], these uncontrolled abnormal movements were not associated with antiparkinsonian medication and did not ameliorate with reduction or cessation of l-DOPA treatment. GIDs were observed in a quarter of the grafted patients in the Freed et al. and half the Olanow et al. trials.

Several issues may have contributed to the onset of GIDs. As patients that received the fetal mesencephalic grafts presented focal points of increased PET signal (Ma et al. 2002), it was proposed that the grafts produced “hot spot” regions in which DA was being excessively released. To answer the question of whether there was an association between GIDs and focal versus widespread distribution of cell grafts in the striatum (Maries et al. 2006), 6-OHDA-treated rats received either a focal striatal transplant of 200,000 cells, or the same number of cells across six different striatal locations. The experiment showed that GIDs were only present in the focal graft recipients. It also brought to light that the number of functional cells within a focal site might be of equal concern. Another proposed contributing factor for GIDs was that the chronic l-DOPA treatment preceding the transplantation may have “primed” the patients to have dyskinesias. In 6-OHDA-intoxicated rats, l-DOPA pre-treatment affected graft integration and functionality, although GIDs were present with or without l-DOPA pre-treatment (Steece-Collier et al. 2009). A study in 24 systemic MPTP-treated vervet monkeys (*Chlorocebus sabaeus*) assessed whether priming the animals to develop l-DOPA-induced dyskinesias (LIDs), and then injecting allogeneic fetal dopaminergic cells in a “spread” or “hotspot” pattern would affect the development of GIDs (Kordower et al. 2016). The investigators did not detect GIDs in any of the monkeys, regardless of cell distribution or condition. Possible species differences could be at play as well as methods of cell preparation. A critical limitation of this report is that the animals’ MPTP-induced parkinsonism spontaneously improved overtime. All the monkeys presented similar mild PD scores with no differences between grafted and control subjects. Thus, the graft’s antiparkinsonian efficacy or graft potential to produce true functional hotspots could not be evaluated.

Inflammation has also been proposed to promote the onset of GIDs through aberrant synapse formation between grafted neurons and host striatal medium spiny neurons [MSNs; (Soderstrom et al. 2008)]. This theory was derived from the observation of GIDs in patients who did not receive immunosuppression following fetal engraftment or those that had been recently taken off an immunosuppressive regimen. Interestingly, the evaluation of spine density maintenance in MSNs through administration of slow-release pellets of the calcium channel antagonist nimodipine has led to intriguing evidence about the importance of preserving MSNs for reducing LIDs and transiently reducing GIDs (Soderstrom et al. 2010). Patient and rodent studies have also highlighted that serotonergic and noradrenergic neurotransmission either by host innervation or by mixed-cell grafts may contribute to GIDs (Shin et al. 2012). As the cause of GIDs is being unraveled, new strategies aiming to prevent or decrease GIDs are being evaluated.

Another potential complication for CBTs was uncovered in 2008, when follow-up postmortem analysis of PD patients treated a decade earlier with fetal grafts found LB-like pathology in the grafted cells (Kordower et al. 2008; Li et al. 2008). Although it is unclear how much the aggregates affected the functionality of the grafts, the implication that PD could be transferred to the grafted cells reverberated throughout the field (see “Parkinson’s disease: then and now”). A preliminary confirmation of α-syn being transferred into grafted cells was obtained in rats injected with adeno-associated viral vector serotype 6 encoding for human α-syn into the striatum after fetal cell engraftment (Kordower et al. 2011).

New clinical trials utilizing fetal mesencephalic tissue for DA cell replacement are currently ongoing in Canada, Europe (Transeuro), South Korea and Mexico (listed in: clinical trials.gov and isrctn.com). The investigators leading these trials aim to optimize the approach by taking advantage of the knowledge gained from the clinical and preclinical studies described above.

## Additional clinical trials using CBTs for DA cell replacement in PD

A number of alternative sources for dopaminergic neurons, including mesencephalic fetal porcine cells, cadaveric human retinal-pigmented epithelium (hRPE) and autologous sympathetic and carotid ganglia, have been investigated to avoid the surgical complication of using autologous adrenal medullary tissue and to overcome the practical and ethical limitations of using human fetal cells for large-scale clinical applications. NHP preclinical studies were only performed for hRPE. The DA-producing cells were attached to gel microcarriers (Spheramine®) and placed into the striatum of parkinsonian monkeys. The grafts improved motor function and postmortem analysis showed cell survival and a mild inflammatory reaction (Watts et al. 2003). Controlled clinical trials for hRPE as well as all the sources listed above failed to show a significant antiparkinsonian effect (Fitzpatrick et al. 2009).

## *Ex vivo* gene therapy for PD

*Ex vivo* gene therapy originated as a method to engineer cells for delivery of therapeutic molecules (Raymon et al. 1997). The cells are typically genetically modified using viral vectors. The main advantage of this method compared to direct intracerebral viral vector delivery (in vivo gene therapy) is that the transfected cells can be monitored before transplantation for the effects of viral infection and the production of a foreign protein. Safety, genetically engineered, “tricks” have been developed to curtail unwanted side effects, such as regulatable promoters to stop gene expression and kill-switches to terminate the cells and completely stop product synthesis.

*Ex vivo* gene therapy strategies have been developed for DA replacement and trophic factor delivery with variable results. MPTP-intoxicated rhesus monkeys received autologous fibroblasts genetically engineered to produce tyrosine hydroxylase [the rate-limiting enzyme for DA production; e.g., (Bankiewicz et al. 2000)] with minimal antiparkinsonian effects. Glial cell line-derived neurotrophic factor (GDNF) producing C2C12 cells were encapsulated and transplanted into MPTP-treated baboons (Kishima et al. 2004) inducing only temporary improvements of their parkinsonian symptoms, probably due to the low survival of encapsulated cells which led to a low and variable protein production. New cell sources for ex vivo gene therapy (see below) are emerging. For example, human neuroprogenitor cells (hNPCs) have been shown to survive and locally produce GDNF in the brain of parkinsonian immunosuppressed rats and monkeys (Behrstock et al. 2006; Emborg et al. 2008). Although the clinical translation for PD has not been pursued (probably due to the poor results of GDNF protein delivery trials; (Richardson et al. 2011)), a clinical trial for amyotrophic lateral sclerosis is currently ongoing (http://www.clinicaltrials.gov).

## Stem cells as sources of cell lines

Biological research breakthroughs and development of new technologies have paved the way for the identification of stem cells (SCs) as new cell sources for regenerative medicine approaches. SCs are defined by their self-renewal capacity and their potential for becoming a different cell type with a specialized function. These properties allow researchers to create cell lines to be repurposed for in vitro studies and transplantation.

hNPCs are typically obtained from the germinal layer of a fetal brain. Although they are not pluripotent SCs (they are already fated toward a brain cell phenotype), in vitro hNPCs can be expanded and differentiated to a DA phenotype (Sanchez-Pernaute et al. 2001). In MPTP-treated monkeys, hNPCs induced improvements of PD signs and postmortem analysis has shown their survival and migration. Their cell progeny seemed to differentiate in vivo into DA-like neurons and glial phenotypes and overall have a “homeostatic” impact (Bjugstad et al. 2005, 2008; Redmond et al. 2007). It should be noted that NPCs have been found in the adult human and NHP brains (Eriksson et al. 1998; Gould et al. 1999). These findings, plus earlier studies in rodents, refuted the idea that the adult brain is incapable of forming new neurons (Altman and Das 1965; Buzanska et al. 2002; Kaplan and Hinds 1977; Kempermann et al. 1997). The option of recruiting resident hNPCs for brain repair has tantalizing possibilities but its utility has not yet been proven (Rakic 2004). Interestingly, one study grafted autologous adult cortical cells (cultured for a couple of weeks) into the caudate of MPTP-intoxicated vervet monkeys. Grafted cells were found 4 months post-surgery and their presence was associated with increased local levels of GDNF (Brunet et al. 2009).

Bone marrow, umbilical cord blood and adult adipose-derived stromal tissue (Fallahi-Sichani et al. 2007; Levy et al. 2008; McCoy et al. 2008) have been proposed as SC sources as they can be obtained for autologous grafts. Although NHP studies with these cells have not been performed, evaluation in rodents suggests that they produce trophic factors that could be beneficial for PD. Several clinical trials are currently ongoing to test the safety and efficacy after administration of mesenchymal stem cells through intravenous, intracarotid or intranasal routes (clinicaltrials.gov). Intravenous infusion of adipose-derived stromal vascular fraction cells is also being investigated for safety and efficacy, as well as for benefits to the quality of daily living in PD patients.

Embryonic stem cells (ESCs) obtained from blastocysts are pluripotent SCs, thus they have the potential to become any cell of the body. In 1995, Thomson et al. (Thomson et al. 1995) reported the isolation of ESCs from rhesus monkeys. Common marmoset ESCs were isolated in 1996 (Thomson et al. 1996), followed in 1998 by human ESCs [hESC; (Thomson et al. 1998)]. Differentiation of ESCs into a DA phenotype was first accomplished in mice (Lee et al. 2000), followed a few years later in human (Perrier et al. 2004; Yan et al. 2005) and rhesus (Takagi et al. 2005). Since then, investigators have been looking for more efficient ways to produce mesencephalic DA neurons, as well as to solve the problems of intracerebral graft survival and other challenges identified by the fetal tissue trials. The ethical dilemma of the cells’ origin triggered the 2001 restriction of USA federal funding for hESC research to studies performed on authorized cell lines, limiting the chances for creating new ones. The 8-year ban on federal funding for ESC research was lifted on March 9, 2009.

Parthenogenesis, somatic cell nuclear transfer and altered somatic cell nuclear transfer have been proposed as alternative sources of pluripotent SCs (Kastenberg and Odorico 2008). In NHPs, parthenogenesis has been used to generate an SC line from cynomolgus monkeys (cyno-1). Parthenogenesis is an asexual form of reproduction; although mammalian eggs cannot fully develop, they can provide blastocysts to generate parthenogenesis-derived ESCs. Using the cyno-1 cell line, investigators differentiated the cells into dopaminergic neurons (Perrier et al. 2004). These cells were successfully transplanted into 6-OHDA-treated rats and one MPTP-treated cynomolgus monkey (Sanchez-Pernaute et al. 2008). hNPCs generated from parthenogenetic hSCs (hpNPCs) have been evaluated in rats and MPTP-lesioned vervet monkeys immunosuppressed with a combination of cyclosporine, prednisone and azathioprine (Gonzalez et al. 2015, 2016). The investigators first demonstrated graft survival and increased DA striatal levels 3 months post-surgery in two NHPs (Gonzalez et al. 2015). Antiparkinsonian efficacy of hpNPCs was then evaluated in 18 monkeys (Gonzalez et al. 2016). The animals were matched according to disability and assigned to one of 3 treatment groups: vehicle (*n* = 6), low (*n* = 6) and high (*n* = 6) cell dosing. Cells were inoculated in the caudate, putamen and substantia nigra. Twelve months post-grafting, the low dose animals showed significant behavioral improvements compared to their baseline condition; however, no significant differences between vehicle and dosing groups were detected. A clinical trial to assess the safety and tolerability of hpNPCs in PD is currently ongoing in Australia (clinical trials.gov). Three patient groups (each to receive different cell number doses) of four patients each who have moderate to severe PD will receive between 30 and 70 million cells injected into the striatum and substantia nigra.

The development of induced pluripotent stem cells (iPSCs) from somatic cells (Takahashi et al. 2007a, b; Yu et al. 2007) has further facilitated the production of additional cell lines for regenerative medicine and disease modeling purposes. iPSCs represent a major advancement towards personalized medicine as cells can be generated from the prospective recipient. iPSCs have been generated from macaque (Deleidi et al. 2011; Liu et al. 2008) and marmoset (Tomioka et al. 2010; Wiedemann et al. 2012; Wu et al. 2010; Vermilyea et al. 2016a) monkeys. The derivation of iPSCs has been modified in recent years using expression plasmids that do not integrate into the host DNA, which increases their safety for clinical translation.

## NHP studies assessing SC-derived DA cell replacement strategies

Over the past decade, several NHP studies have analyzed the potential use of SCs from different sources and at different stages of differentiation for DA cell replacement (Table 2).

**Table 2:** Peer-reviewed publications in NHP models of PD evaluating SCs as sources for dopaminergic cell replacement

References	Host species	Cell species; graft type	Cell type	Cell labeling	Transplant location	Model	*n*	Immunosup.	l-DOPA	Behavioral test	PET imaging	Duration
Takagi et al. (2005)	Cynomolgus (*M. fascicularis*)	Cynomolgus; allograft	ESC-derived DAergic progenitors	BrdU	Bilateral putamen; 3 tracts per side, each with 4 deposits	MPTP i.v. (0.4 mg/kg; twice weekly for 7 weeks)	6	CsA 10 mg/kg	None	Clinical rating	[^18^F]DOPA	14 weeks
	Cynomolgus (*M. fascicularis*)	N/A	Sham-operated	N/A	N/A	MPTP i.v. (0.4 mg/kg; twice weekly for 7 weeks)	4	CsA 10 mg/kg	None	Clinical rating	[^18^F]DOPA	14 weeks
Kikuchi et al. (2011)	Cynomolgus (*M. fascicularis*)	Human; xenograft	iPSC-derived DAergic progenitors	None	Bilateral putamen (d28 to right, d42 to left); 6 tracts per side, each with 4 deposits	MPTP i.v. (0.4 mg/kg; twice weekly until symptoms persisted)	1	FK506 0.05 mg/kg	None	Clinical rating, spontaneous movements, Gross Movements, Raisin pick-up test	[^18^F]DOPA, [^11^C]DTBZ, [^11^C]PE2I, [^18^F]FLT	6 months
Daadi et al. (2012)	Green monkey (*C. sabaeus*)	Human; xenograft	ESC-derived DAergic progenitors	GFP	Caudate and SN	MPTP i.m. (2.25 mg/kg cumulative dose; administered over 5-day period)	4	CsA 5 mg/kg/day Prednisone 2 mg/kg tapering to 0.6 mg/kg/day Azathioprine 5 mg/kg/day reduced to 1 mg/kg/day	None	None	None	2 months
Kriks et al. (2011)	Rhesus (*M. mulatta*)	Human; xenograft	ESC-derived DAergic progenitors	GFP	Bilateral posterior caudate, pre-commissural putamen and overlying white matter (GFP to one side, unmarked to other); 3 tracts per side	MPTP ICA (3 mg) followed by weekly i.v. administration (dose n.r.)	2	CsA 30 mg/kg tapered to 15 mg/kg	None	None	None	1 month
Emborg et al. (2013b)	Rhesus (*M. mulatta*)	Human; xenograft	ESC-derived DAergic progenitors	GFP	Unilateral (ipsi-MPTP) rostral, medial and caudal caudate and putamen, and SN; 7 tracts total	MPTP ICA (3 mg)	3	CsA 40–50 mg/kg	None	None	None	3 months
Emborg et al. (2013a)	Rhesus (*M. mulatta*)	Rhesus; autograft	iPSC-derived DAergic progenitors	GFP	Unilateral (ipsi-MPTP) precommisural and commissural caudate, pre-commisural, commissural and postcommisural putamen, and SN; 6 tracts total	MPTP ICA (3 mg)	3	None	None	None	None	6 months
Wakeman et al. (2014b)	Green monkey (*C. sabaeus*)	Human; xenograft	ESC-derived DAergic progenitors	GFP	Caudate and SN	MPTP i.m. (2 mg/kg cumulative dose; administered over 5-day period)	2	Azathioprine, CsA and prednisolone	None	None	None	6 weeks
Hallett et al. (2015)	Cynomolgus (*M. fascicul ris*)	Cynomolgus; autograft	iPSC-derived DAergic progenitors (Cooper et al. 2010 protocol)	None	Unilateral postcommissural putamen; 4 tracts total	MPTP i.v. (0.15–0.3 mg/kg) every 1–2 weeks	1	None	None	Clinical rating, activity monitoring, FMS	[^11^C]CFT	2 years
	Cynomolgus (*M. fascicularis*)	Cynomolgus; autograft	iPSC-derived DAergic progenitors (Sundberg et al. 2013 protocol)	None	Unilateral postcommissural putamen; 4 tracts total	MPTP i.v. (0.15–0.3 mg/kg) every 1–2 weeks	2	None	None	Clinical rating, activity monitoring, FMS	[^11^C]CFT	2 years
	Cynomolgus (*M. fascicularis*)	Cynomolgus; allograft	ESC-derived DAergic progenitors (Sanchez-Pernaute et al. 2005 protocol)	None	Unilateral putamen	MPTP i.v. (0.15–0.3 mg/kg) every 1–2 weeks	3	None	None	Clinical rating, activity monitoring, FMS	[^11^C]CFT	1 year
	Cynomolgus (*M. fascicularis*)	N/A	N/A	N/A	N/A	MPTP i.v. (0.15–0.3 mg/kg) every 1–2 weeks	4	None	None	Clinical rating, activity monitoring, FMS	[^11^C]CFT	2 years
Wang et al. (2015)	Cynomolgus (*M. fascicularis*)	Cynomolgus; autograft	iPSC-derived DAergic progenitors	GFP and Feridex IV	Unilateral (ipsi-MPTP) rostral, intermediate and caudal caudate and putamen, and SN; 7 tracts total	MPTP ICA (3 mg)	1	None	None	Clinical rating	None	6 months
	Cynomolgus (*M. fascicularis*)	N/A	Vehicle	N/A	Unilateral (ipsi-MPTP) rostral, intermediate and caudal caudate and putamen, and SN; 7 tracts total	MPTP ICA (3 mg)	3	None	None	Clinical rating	None	6 months

*Immunosup* immunosuppression, *PET* positron emission tomography, *ESC* embryonic stem cell, *iPSC* induced pluripotent stem cell, *DAergic* dopaminergic, *BrdU* 5-Bromo-2′-deoxyuriding, *MPTP* 1-methyl-4-phenyl-1,2,3,6-tetrahydropyridine, *L-DOPA* L-3,4-dihydroxyphenylalanine, *ipsi* ipsilateral, *i.v.* intravenous, *ICA* intracarotid artery, *mg* milligram, *kg* kilogram, *CsA* Cyclosporine A, *GFP* green fluorescent protein, *n.r.* not reported, *N/A* not applicable, *FMS* fine motor skills, *SN* substantia nigra, *[18 F]DOPA* 6-[18 F]fluoro-l-3,4-dihydroxyphenylalanineresentatio, *[11 C]DTBZ* [11 C]dihydrotetrabenazine, *[11 C]PE2I* (*E*)-*N*-(3-iodoprop-2-enyl)-2β-carbo[11 C]methoxy-3β-(4-methylphenyl)nortropane, *[18 F]FLT* 3′-deoxy-3′3[18 F]fluorothymidine

Takagi et al. investigated monkey ESC-derived neural progenitors capable of producing dopaminergic neurons for transplantation into an NHP model of PD (Takagi et al. 2005). ESCs from a cynomolgus monkey (*Macaca fascicularis*) were differentiated on stromal cells with the addition of FGF2 and FGF20, and labeled in vitro with BrdU for postmortem identification. The ESC-derived dopaminergic progenitor cells were then transplanted into systemic MPTP-treated cynomolgus monkeys. Beginning at 10 weeks post-brain surgery, the neurological parkinsonian scores of animals receiving grafts (*n* = 6) decreased significantly (*p* < 0.05) compared with sham controls (*n* = 4) and were associated with a significant increase in striatal [^18^F]fluorodopa uptake observed by in vivo PET. Postmortem analysis at 14 weeks post-grafting verified survival of BrdU/TH colabeled neurons. Neither mitosis (identified by Ki67-immunoreactivity) nor tumor formation was observed in animals that received ESC-derived neuronal transplants.

Aiming for human translation, Kriks et al. differentiated floorplate-derived dopaminergic neurons from human ESCs (hESCs), labeled half of the cells with GFP and then transplanted the labeled cells into one side of the striatum, and unlabeled cells to the other, of two systemic MPTP-treated rhesus monkeys immunosuppressed by CsA administration (Kriks et al. 2011). At one-month post-transplantation, grafted cells were observed in the posterior caudate and pre-commissural putamen as well as Iba1+cells, suggesting immunoreaction by the host. Daadi et al. 2012 and Wakeman et al. 2014 tested hESC-derived DAergic neurons expressing GFP in the caudate and nigra of MPTP-intoxicated vervet monkeys immunosuppressed with cyclosporine, prednisone and azathioprine (Daadi et al. 2012; Wakeman et al. 2014b). The 4 monkeys treated by Daadi et al. showed a few TH-positive grafted cells, extending neurite outgrowth and expressing synaptic markers 2 months post-surgery; cell counts of grafts and immunological response were not reported. In the Wakeman et al. study, the two monkeys presented co-expression of GFP and βIII-tubulin positive cells but not TH, dopamine transporter (DAT) or other markers of midbrain floorplate differentiation 6 weeks after grafting, suggesting de-differentiation; evaluation of inflammatory markers was not reported. Emborg et al. also studied hESC-derived DAergic neurons; however, the brain evaluations were performed 3 months after grafting. The cells were genetically engineered to express GFP and grafted in the striatum and nigra of three rhesus monkeys that received MPTP by carotid artery injection. The three animals were immunosuppressed by daily oral dosing of cyclosporine that was started 48 h prior to grafting (Emborg et al. 2013b). Postmortem analysis at 3 months revealed graft survival in only one of the three monkeys. The graft was infiltrated with GFAP, CD68 and CD45 immunoreactive cells, suggesting ongoing immune reaction despite immunosuppression. These results further demonstrated that immunological issues are a major concern for xenografts and that allogeneic or autologous transplants may render better graft survival and integration.

Grafts of human iPSC (hiPSC)-derived dopaminergic neurons have also been attempted. In 2011, Kikuchi et al. reported their evaluation of grafting into one FK506 immunosuppressed MPTP-treated monkey (Kikuchi et al. 2011). The hiPSCs were differentiated into DAergic neural progenitors in feeder-free conditions but were not labeled. Day 28 (d28) and d42 neurospheres were transplanted into the right and left putamen, respectively. Graft size and function was evaluated at 1, 3 and 6 months by MRI and at 6 months by PET using the radioligands 6-[^18^F]fluoro-l-3,4-dihydroxyphenylalanine ([^18^F]DOPA), [^11^C]dihydrotetrabenazine ([^11^C]DTBZ), (*E*)-*N*-(3-iodoprop-2-enyl)-2β-carbo[^11^C]methoxy-3β-(4-methylphenyl)nortropane ([^11^C]PE2I), to assess for DA synthesis, vesicle transport, and DA reuptake, and with 3′-deoxy-3′3[^18^F]fluorothymidine ([^18^F]FLT) to visualize cell proliferation. Interestingly, MRI showed increase in graft size on the side of grafted d28 neurospheres compared to d42. In general, PET did not identify any meaningful graft-related uptake, with exception of increased binding of ([^11^C]PE2I) in the d42-grafted putamen, suggesting cell differentiation. Neurological evaluation throughout the study showed no behavioral recovery. Postmortem analysis 6 months after transplantation revealed graft survival; d42 spheres produced higher amounts of TH+cells compared with the d28 spheres, while also maintaining some progenitor populations, which explains the observed intracerebral graft growth; immunological response was not reported.

**Fig. 2:**
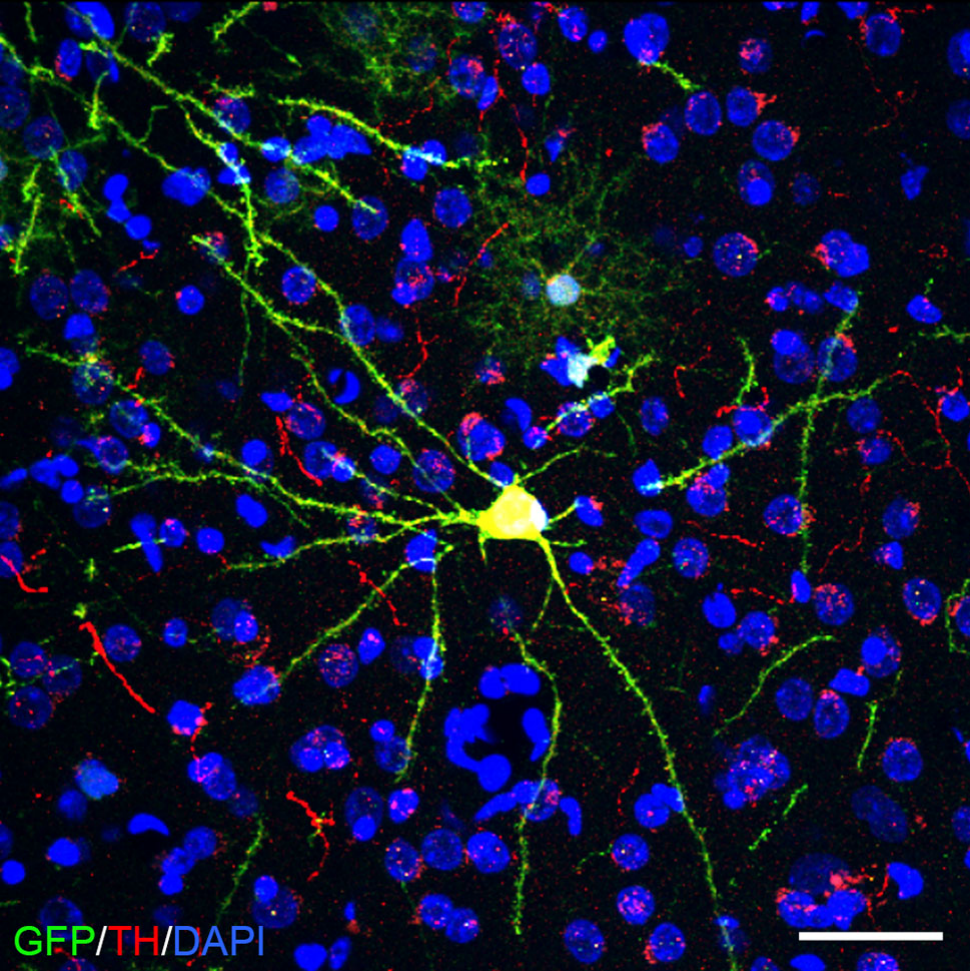
An example of a grafted autologous rhesus IPSC-derived dopaminergic neuron labeled with GFP (*green*) and immunostained against TH (*red*); DAPI (*blue*). *Scale bar* 50 μm

A major advantage of iPSC technology is that patient-specific cells can be generated for grafting, which minimizes host immune reaction and avoids immunosuppression. In 2013, Emborg et al. reported their findings in three rhesus monkeys that received an intracarotid artery injection of MPTP, followed 6 months later with autologous iPSC-derived neuroprogenitors into the striatum and nigra, without immunosuppression [Fig. 2; (Emborg et al. 2013a)]. For identification, the cells were genetically modified to express GFP. Six months post-surgery, brain analysis showed abundant GFP-positive neuron-like cells, which integrated with the host brain and expressed TH. Most importantly, infiltration of host immune cells observed by CD3 and CD8 reactivity was minimal while HLA-DR and GFAP were mild, similar to MPTP-induced inflammation. Although neurological scores were not reported, it was mentioned that the animals’ PD motor signs did not improve; the lack of recovery was probably due to low numbers of DAergic neurons grafted. Shortly after, Morizane et al. compared autologous and allogeneic transplantation of iPSC-derived neural cells in intact cynomolgus monkeys (Morizane et al. 2013). As expected, the allogeneic transplants elicited a marked immune response observed, with in vivo PET, as increased uptake of [^11^C]PK11195 compared to the autografts, further emphasizing the benefit of self-derived cells for translational applications.

In 2015, Hallett et al. reported that the differentiation protocols used to generate iPSC-derived DAergic neurons could be a factor for survival and functional integration of grafted cells (Hallett et al. 2015). After ten cynomolgus monkeys were rendered stably parkinsonian through systemic MPTP administration, the animals received iPSC- or ESC-derived DAergic grafts differentiated following three different protocols: (Cooper et al. 2010) (*n* = 1), (Sundberg et al. 2013) (*n* = 2), and a modified (Sanchez-Pernaute et al. 2005) (*n* = 3; for ESCs); no transplanted animals (*n* = 4) were used as controls. The grafts derived from the Sanchez–Pernaute differentiation protocol did not survive and were thus separately analyzed as the non-surviving transplant group, compared with the Cooper- and Sundberg-derived grafts. Only the animal that received cells using the Cooper differentiation protocol presented functional recovery of daytime activity and movement analysis. The animal recovered to pre-MPTP levels for global activity, and improved in fine motor skills after 2 years. [^11^C]CFT PET imaging corroborated graft function with increased uptake around the transplant site. The lack of functional recovery and survival of the Sundberg and Sanchez-Pernaute grafts provide evidence that DAergic differentiation patterning may be critical for functional integration.

Wang et al. also assessed the feasibility of autologous iPSC-derived DAergic neuron grafts to one MPTP-treated cynomolgus monkey (Wang et al. 2015). Throughout the 6-month survival period, only between 6–8, and 22–24 weeks, the transplant monkey appeared to have an improved clinical rating compared with the control monkeys (*n* = 3). Postmortem analysis in the transplanted monkey showed graft survival and TH immunoreactivity.

An examination of these SC-based studies highlights that, similar to the fetal tissue reports in NHPs, they were mainly feasibility/safety experiments, performed in a few animals (with exception of Takagi et al. 2005) and focusing on assessing cell survival, cell proliferation/tumor formation and whether the grafted cells integrated and showed a DA phenotype. Some antiparkinsonian effects were described. Considering the ongoing improvements on SC research, this approach shows responsible use of the NHP resource and also underscores that the results should be kept in perspective with the limitations of the experimental design and outcome measures utilized.

## Nigral vs. striatal targets

**Fig. 3:**
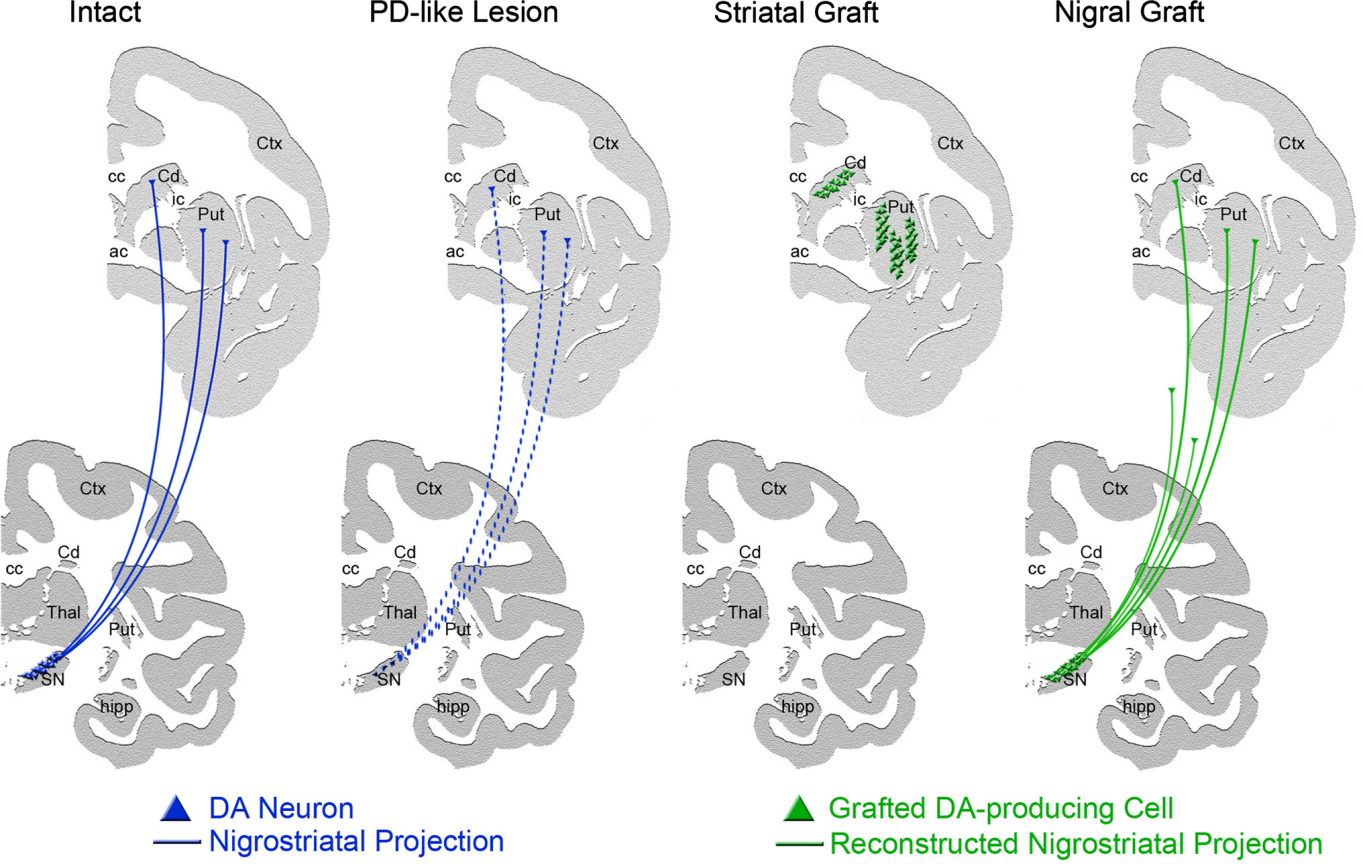
Graphical depiction of a rhesus monkey brain hemisphere in intact and PD-like conditions and with striatal and nigral grafts. The main area of projection of nigral dopaminergic neurons is the striatum, which is composed by the caudate nucleus and putamen. In PD and PD-like conditions (e.g.: after MPTP intoxication), nigral dopaminergic neurons die; thus, striatal dopamine (DA) is decreased. Grafting of DA-producing cells into the striatum is envisioned as a way to ensure DA availability in the area of projection. Nigral grafts are proposed as a way to reconstruct the nigrostriatal pathway. *Cd* caudate, *ic* internal capsule, *Put* Putamen, *ac* anterior commissure, *cc* corpus callosum, *Ctx* cortex

For DA cell replacement, the field strives to recreate and purify A9 DA nigral cells, yet in patients, the cells have been mainly transplanted in the postcommisural putamen [see review: (Redmond 2002)] with a few exceptions in which the fetal nigral cells were placed in the nigra and putamen [e.g.: (Mendez et al. 2002); Fig. 3]. Putaminal targeting aims to provide DA in the area where needed, while nigral grafting is proposed to restore the nigrostriatal pathway, including the regulation of grafted cell activity and restoring the DA tone in the substantia nigra pars reticulata.

Theoretically, the nigral location is a most optimal method for brain repair, yet an obstacle for its application is the long distance to be covered by the axons of cells inoculated in the nigra to reach their striatal targets. The successful reconstruction of the nigrostriatal pathway requires time for axonal extension, axonal guidance and ultimately recognition and synaptic connection with targets. In rodents, the feasibility and advantages of the approach have been reported using fetal cells (Mendez et al. 1996) and ESCs (Grealish et al. 2014), observing positive behavioral effects as soon as 6 weeks post-fetal grafting and 18 weeks with ESC-derived DA cells, with the latter requiring more time to mature and integrate in the host. In NHPs and humans, the nigrostriatal distance is greater, which means that the time needed after grafting to observe behavioral benefits may also increase; therefore, multisite grafting has been preferred.

The feasibility of grafting fetal midbrain cells in the nigra (Collier et al. 2002) or in combination with fetal striatal grafts for axonal guidance (Sladek et al. 2008) has been demonstrated in MPTP-treated vervet monkeys; in both studies axonal extension through the nigrostriatal pathway and some modest caudal putamen reinnervation was observed at 6 months; although follow-up studies of co-grafting fetal nigra and striatum had limited effect (Redmond et al. 2009). Localized delivery of trophic factors has been proposed as a method to stimulate and guide axonal growth. Striatal GDNF overexpression via AAV2 vectors was reported to enhance the survival and outgrowth of fetal dopamine neurons implanted in the striatum (Elsworth et al. 2008). A follow-up comparison between MPTP-intoxicated monkeys treated with AAV5 GDNF in the caudate nucleus or solid fetal midbrain grafts in the caudate and putamen or a combination of AAV-GDNF and fetal grafts or buffered saline solution did not reveal greater functional improvement in the AAV-GDNF and fetal grafts monkeys during the 8 months of observation (Redmond et al. 2013). AAV2-induced overexpression of GDNF in the caudate supported outgrowth of fetal midbrain grafts maximally observed at 22 months post-grafting (Redmond et al. 2009), as well as stimulated neurite extension and dopaminergic differentiation of hNC grafts, both placed in the nigra of MPTP monkeys (Wakeman et al. 2014a). The studies suggest that reconstruction of the nigrostriatal pathway can be achieved and that research on methods aiming to promote and guide axonal growth is needed.

## The future of CBTs and NHP studies

NHP studies have paved the way for the first clinical trials using CBTs for PD. Although the number of monkey experiments and/or total number of animals was a fraction compared to rodent studies, they provided key evidence that CBTs could be used to treat PD. NHP experiments have facilitated the optimization of cell collection and preparation methods, cell viability, as well as identify potential brain targets. Today, old approaches are being re-evaluated and optimized and new ones are being developed; some are being translated into the clinic (Barker 2014). NHP research can provide an even more crucial insight into CBTs, but as a limited resource, prioritization of issues to be evaluated will be critical. How cells are prepared and stored affect engraftment, the methods of delivery, the choice of targets and related timelines of recovery are basic questions that still need to be solved and tested in NHPs to improve CBT outcome. The response of the host to the candidate CBT encompasses a different set of topics related to efficacy, safety (e.g.: prevention of GIDs), immunomodulation and inflammatory response and propagation of α-syn. Each of these issues can also benefit from careful NHP research.

Several groups are invested in the development of the optimal cell for transplantation, working on generating cell lines of “superdonors” with immunological compatibility and improving the methods of cryoprotection to facilitate clinical application, as creating a cell line per individual to be treated would be a daunting task. While the idea of testing in NHPs the same cell lines to be grafted to humans is compelling, current data presented in this review highlight the limitations of the xenograft approach with current immunosuppression paradigms. Thus, a more parsimonious approach would be to produce equivalent cells derived from the same species, at least until the NHP equivalent of severe combined immune deficiency (SCID) mice becomes available (Sato et al. 2016).

**Fig. 4:**
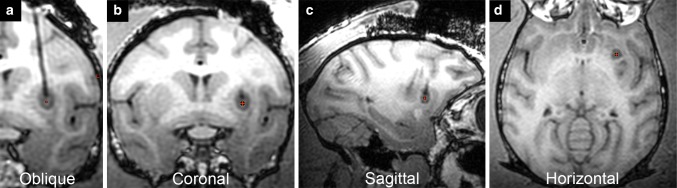
Real-time intraoperative MRI for intracerebral delivery of stem cells provides feedback of cannula placement and infusion site: **a** *oblique*, **b** *coronal*, **c** *sagittal* and **d** *horizontal planes*

Due to the brain volume and complexity of NHPs, questions regarding intracerebral targets and graft distribution will benefit from NHP studies and noninvasive imaging approaches. For example, great strides have been made towards the development and improvement of intraoperative MRI (iMRI) methods (Mislow et al. 2009). Silvestrini et al. (Silvestrini et al. 2015) used real-time iMRI (RT-iMRI) for cell transplantation into a swine and cadaveric human head as a concept for application in the human brain. The platform technology utilizes a radially branched deployment strategy to access multi-directional deposit sites along a single cannula insertion tract. Our group modified an RT-iMRI delivery system that has a pivot point base, a clear silica cannula and inline pressure monitoring system (Emborg et al. 2010, 2014) for the in vivo delivery of DAergic progenitor cell spheres into the putamen of a rhesus monkey [Fig. 4; (Vermilyea et al. 2016b)]. Malloy et al. (2016) used an MRI-compatible delivery system for MRI monitoring of the distribution of cells pre-labeled with a contrast agent into a baboon basal ganglia. These new MRI-based imaging methods can increase the safety and accuracy of the grafting procedures and facilitate the evaluation of different targeting sites. In that regard, imaging technologies, such as diffusion tensor imaging (Hall et al. 2016), can be applied to preclinical studies to evaluate circuit reconstruction, complementary to traditional PET imaging with DA-related radioligands. Application of imaging methods overtime to monitor graft integration and function would be critical to reduce the number of NHPs groups needed per experiment to understand recovery timelines.

At this time, GIDs cannot be prevented or cured; NHP safety studies on methods aiming to modulate grafted cell activity are needed. Optogenetics is a technique that uses light to control cells that have been genetically modified to express light-sensitive ion channels (Lerner et al. 2016). Optogenetic approaches have been applied to control electrophysiological and neurochemical properties of grafted SC-derived DA neurons in rodent models (Chen et al. 2015; Steinbeck et al. 2015), yet its clinical translation is not recommended as the patients would require the intracerebral placement of a probe to locally deliver the appropriate light wavelength. Compared to optogenetics, the designer receptors exclusively activated by designer drugs (DREADDs) technology use noninvasive methods to exert its effects, as it depends on designer drugs to modulate the activity of cells that are genetically modified to express the corresponding receptor. DREADDs have been used to modulate human PSC-derived DA neurons (Chen et al. 2016). There has been a lot of discussion regarding CBT safety centered on defining an adequate cell number and volume for intracerebral delivery. The use of DREADDs or an equivalent technology would allow more flexibility as, theoretically, cells can be further excited if behavioral recovery is not observed, or inhibited if serious GIDs occur. Basically, it would provide a strategy for patient-specific graft modulation that could be monitored by a combination of clinical and PET imaging tools.

How can the value of the next generation of NHP studies be maximized for the successful clinical translation of CBTs for PD? We propose that first and foremost, the investigators should use NHP PD models that match the question at hand (Table 1). As we previously discussed, until now all monkey studies of CBTs for PD have been performed in neurotoxic models. Many basic questions regarding feasibility, efficacy, and safety can be answered in these models. Yet, the same way that only old animals can provide insight on grafting in the aging brain, studies in models of synucleinopathies are needed to assess the impact of protein aggregation in graft efficacy. In that regard, the field is anxiously waiting for the validation of current genetic models and the availability of transgenic and genomic edited PD monkeys. Second, a well-designed feasibility study in a few monkeys presents an opportunity to learn if an approach is worth pursuing, with the caution that improvement of PD symptoms could be due to individual variability or spontaneous recovery. Demonstration of efficacy requires properly powered NHP experiments with blind group assignment and evaluation. Third, the surgical method of cell delivery is critical to ensuring appropriate targets, optimized cell survival and distribution and should not be minimized. Fourth, the animals should be assessed with multiple outcome measures with clinical impact to maximize the knowledge to be extracted from the study. Behavioral evaluations complemented by in vivo imaging methods can facilitate postsurgical follow-up, especially for CBT studies taken several years to be completed. Postmortem analysis should include unbiased cell counts and evaluation of host immunological response in order to inform about the safety, efficacy and limitations of the approach. Fifth, grafted cells should be pre-labeled to facilitate identification from host cells. Images of grafts should be in low and high magnification to assess the extent of cell survival and interaction with host. Sixth, there are no bad results, but poorly designed experiments. Publication of positive as well as negative experimental results in NHPs should be encouraged for the overall evolution of the field.

To conclude, NHP PD research plays a small but critical role in CBT clinical translation. Ultimately, investigators should remember that whether CBTs for PD work will depend on the benefits outweighing the patients’ risk and that NHPs have unique characteristics to help identify and solve these problems.
